# Efficacy of initial caspofungin plus trimethoprim/sulfamethoxazole for severe PCP in patients without human immunodeficiency virus infection

**DOI:** 10.1186/s12879-023-08372-z

**Published:** 2023-06-16

**Authors:** Hui Qi, Danjiang Dong, Ning Liu, Ying Xu, Mengzhi Qi, Qin Gu

**Affiliations:** Department of Intensive Care Unit, The Affiliated Nanjing Drum Tower Hospital of Nanjing University Medical School, Nanjing, 210008 China

**Keywords:** Pneumocystis pneumonia, Trimethoprim–sulfamethoxazole, Caspofungin, Combination therapy

## Abstract

**Background:**

The number of pneumocystis pneumonia (PCP) cases is increasing in immunocompromised patients without human immunodeficiency virus infection (HIV), causing serious morbidity with high mortality. Trimethoprim/sulfamethoxazole (TMP/SMZ) monotherapy has limited effectiveness in the treatment of PCP. Clinical data on whether initial caspofungin plus TMP/SMZ for this disease is superior to monotherapy in non-HIV-infected patients are limited. We aimed to compare the clinical effectiveness of these regimens for severe PCP in non-HIV patients.

**Methods:**

A retrospective study reviewed 104 non-HIV-infected patients with confirmed PCP in the intensive care unit between January 2016 and December 2021. Eleven patients were excluded from the study because TMP/SMZ could not be used due to severe hematologic disorders or clinical data were missing. All enrolled patients were divided into three groups according to different treatment strategies: Group 1 received TMP/SMZ monotherapy, Group 2 received caspofungin combined with TMP/SMZ as first-line therapy, and Group 3 initially received TMP/SMZ monotherapy and later received caspofungin as salvage therapy. The clinical characteristics and outcomes were compared among the groups.

**Results:**

A total of 93 patients met the criteria. The overall positive response rate of anti-PCP treatment was 58.06%, and the overall 90-day all-cause mortality rate was 49.46%. The median APACHE II score was 21.44. The concurrent infection rate was 74.19%, among whom 15.05% (n = 14) of those patients had pulmonary aspergillosis, 21.05% (n = 20) had bacteremia, and 23.65% (n = 22) had CMV infections. The patients who received initial caspofungin combination with TMP/SMZ had the best positive response rate (76.74%) compared to others (*p* = 0.001). Furthermore, the group that received initial caspofungin combined with TMP/SMZ had a 90-day all-cause mortality rate (39.53%) that was significantly different from that of the shift group (65.51%, *p* = 0.024), but this rate showed no statistically significant difference compared with that in the monotherapy group (48.62%, *p* = 0.322). None of the patients had serious adverse events from caspofungin therapy.

**Conclusions:**

For non-HIV-infected patients with severe PCP, initial combination therapy with caspofungin and TMP/SMZ is a promising first-line treatment option compared with TMP/SMZ monotherapy and combination therapy as salvage therapy.

## Introduction

The number of cases of pneumocystis pneumonia (PCP) has increased in immunocompromised patients without HIV in recent decades, causing serious morbidity with high mortality [1, 2]. The first-line therapy of trimethoprim-sulfamethoxazole (TMP/SMZ) has been shown to be an effective regimen in patients, especially when focusing on HIV-infected patients. However, only a small number of observational studies [3–5] and one comparative randomized trial [6] have shown efficacy in non-HIV-infected patients. Therefore, a substantial proportion of individuals experience perceived treatment failure, and the optimum treatment of non-HIV patients remains challenging. Alternative drugs, including caspofungin, have been increasingly scrutinized for their efficacies in treating PCP [7]. Previous studies reported that caspofungin combined with TMP/SMZ could possibly create synergy for this disease. Caspofungin has shown similar efficacy to TMP-SMZ in improving survival and reducing pulmonary edema and cyst burden in animal models [8]. To date, the current clinical data on caspofungin combined with TMP/SMZ in the treatment of PCP have reported divergent findings [9–11]. The efficacy of echinocandins in the treatment or prevention of human PCP remains controversial and is still being explored. Limited literature has focused on the efficacy in the non-HIV population [12].

In this retrospective study, we aimed to examine the clinical effectiveness and safety of caspofungin plus TMP/SMZ as a first-line therapy for moderate-to-severe PCP in non-HIV patients.

## Methods

We retrospectively reviewed the medical records of adult patients diagnosed with severe PCP in our institute between January 2016 and December 2021. The diagnostic criteria for PCP were as follows: Pneumocystis carinii encapsulated in qualified sputum specimens and bronchoalveolar lavage fluid (BALF) stained with hexamine silver and a PCR-based assay were considered confirmatory in patients. The definition of severe PCP are as follows according to Miller [13] criteria: (1) acute onset of dyspnea or tachypnoea at rest, persistent fever and cough; (2) diffuse interstitial infiltration of both lungs with ground glass changes on chest X-ray or CT; (3) arterial partial pressure of oxygen (PaO2) < 60 mmHg at rest when breathing room air or arterial oxygen saturation(SaO2) < 91% when breathing room air.

Patients were excluded if (1) they had confirmed coinfection with HIV; (2) they were younger than 18 years of age; or (3) TMP/SMZ treatment could not be used due to severe hematologic disorders.

The study was approved by the ethics committee (2021-594-02).

The patients were divided into three groups according to their therapeutic regimens. In group 1, the patients received standard PCP therapy, which consisted of TMP/SMZ as monotherapy. The patients in group 2 received cospofungin with TMP/SMZ initially when starting anti-PCP treatment. In Group 3, the patients initially received TMP/SMZ monotherapy and later received caspofungin as salvage therapy.

All data were retrieved from the patients’ electronic and physical medical records. Patients’ information included data regarding clinical symptoms and laboratory test results on admission; underlying diseases; radiological images; acute physiology and chronic health evaluation (APACHE) II score on the day of admission to ICU; duration of PCP treatment and the PCP treatment regimen administered.

The primary outcomes of the study were the positive response rate and 90-day all-cause mortality rate. A positive response was defined as follows [14–16]: ① clinical cure or showing definitive clinical improvement with baseline signs, resolution of dyspnea and chest infiltrates; ②clinical improvement sustained at least 2 to 4 weeks after cessation of antifungal therapy.

### Statistical analysis

We used descriptive statistics to present the patients’ baseline characteristics in each group. Categorical variables are presented as counts (percentages) and were compared using the χ2 test, and continuous variables were compared using one-way ANOVA. To identify independent risk factors, parameters with *P* < 0.05 in the univariate analysis were analyzed using a multivariate logistic regression model. The Kaplan–Meier curve and log-rank test were used for survival analysis. Two-tailed P values were adopted, and *P* < 0.05 was considered significant. All statistical analyses were performed using SPSS statistical software v.20.0 (IBM, Chicago, IL, USA).

## Result

### Baseline characteristics

Data of 104 patients who met the inclusion criteria were collected between January 2016 and December 2021. 6 patients were excluded due to clinical data loss and 7 patients were excluded because of TMP/SMZ not being used due to severe hematologic disorders. A total of 93 patients were involved in the study. Overall baseline characteristics stratified by therapeutic regimens are presented in Table 1. The median age of these patients was 54.54 ± 15.77 years old, and 39.78% of them were male. The median APACHE II score was 21.44. The concurrent infection rate was 74.19%, among whom 15.05% (n = 14) of these patients had pulmonary aspergillosis, 21.05% (n = 20) had bacteremia, and 23.65% (n = 22) had CMV infections. The proportion of the patients included in the study with shock was 35.48%, and the proportion requiring invasive mechanical ventilation support was 43.01%. The median time from symptom onset to PCP treatment was 7 (4–9.5) days. The median time from symptom onset to ICU admission was 8.0 (5.0–13.5) days. The average duration of treatment with TMP/SMZ was 13.8 ± 5.7days; whereas systemic use of glucocorticoids was 83.9%(n = 78). The duration of treatment TMP/SMZ combined with caspofungin was 13.9 ± 6.8 days and 10.9 ± 5.3 days in the group 2 and group3 respectively.

**Table 1:** Overall baseline characteristics in the cohort stratified by therapeutic regimen

variables	All(n = 93)	Group1(n = 21)	Group2(n = 43)	Group3(n = 29)	p value
Male [n (%)]	37(39. 8%)	7(33.3%)	12(27.9%)	11(37.9%)	0.667
Age(years), mean ± SD	54.5 ± 15.8	58.4 ± 16.3	51.4 ± 14.3	56.4 ± 17.0	0.191
APACHII score	21.4 ± 5.9	22.1 ± 5.9	20.7 ± 5.6	22.3 ± 6.3	0.509
Underlying diseases, n (%)					
Rheumatic diseases	52(55.9%)	10(47.6%)	25(58.1%)	17(58.6%)	0.684
Organ transplantation	5(5.4%)	1(4.8%)	3(6.9%)	1(3.4%)	0.801
tumors	14(15.1%)	2(9.5%)	9(20.9%)	3(10.3%)	0.339
Others*	22(22.6%)	8(33.3%)	6(14.0%)	8(27.6%)	0.086
Lab examination					
WBC (×10^9^/L)	9.7 ± 6.9	12.3 ± 7. 7	8. 7 ± 6.2	9.4 ± 7.0	0.150
Neu (×10^9^/L)	8.1 ± 5.6	9.4 ± 5.9	7.0 ± 4.2	8.7 ± 7.0	0.200
Lym (×10^9^/L)	0.7 ± 0.9	0.7 ± 0.5	0.7 ± 1.2	0.6 ± 0.7	0.682
Plt (×10^9^/L)	163.3 ± 108.6	156.6 ± 96.1	170.2 ± 125.8	157.8 ± 91.3	0.852
CD4/CD8	1.1 ± 0.8	1.2 ± 0.5	1.2 ± 0.9	1.0 ± 0.8	0.723
PCT (ng/ml)	0.40[0.13,1.54]	0.6[0.1,3.6]	0.4[0.1,1.5]	0.4[0.2,0.9]	0.956
CRP (mg/l)	105.8 ± 82.7	111.4 ± 108.2	97.5 ± 77.1	113.9 ± 70.7	0.670
Serum BDG (ng/l)	856.0 ± 759.9	646.3 ± 542.1	956.2 ± 850. 8	870.2 ± 867.2	0.354
LDH(U/L)	1187.7 ± 669.9	1187.0 ± 806.2	1104.7 ± 627.5	1311.3 ± 626.9	0.444
Urea nitrogen(mmol/l)	11.3 ± 8.4	11.1 ± 6.7	11.9 ± 10.2	10.6 ± 6.4	0.801
Creatinine (µmol/L)	67.0[44.0,124.0]	75[ 46.6,114.0]	63[44.0,169.0]	57[43.9,108.5]	0.204
PaO2/FiO2 (mmHg)	113.0 ± 53.1	118.9 ± 54.4	114.3 ± 55.6	106.9 ± 49.4	0.721
Coinfections, n (%)					
Viruses	22(23.7%)	5(23.8%)	12(27.9%)	5(17.2%)	0.579
Bacteremia	20(21.5%)	5(23.8%)	5(11.6%)	10(34.5%)	0.066
Pulmonary aspergillosis	14(15.1%)	7(33.3%)	2(4.6%)	5(17.2%)	0.010
Radiographic findings, n (%)					
Bilateral GGOs	70(75.3%)	16(76.2%)	31(72.1%)	23(79.3%)	0.780
Pleural effusion	19(20.4%)	3(14.3%)	11(25.6%)	5(17.2%)	0.504
Pneumothorax	4(4.3%)	1(4.8%)	1(2.3%)	2(6.9%)	0.640
Treatment					
Systemic use of glucocorticoids, n (%)	78(83.9%)	15(71.4%)	38(88.4%)	25(86.2%)	0.206
Duration of TMP/SMZ, days	13.8 ± 5.7	15.7 ± 6.2	13.8 ± 6.7	15.2 ± 5.1	0.446
Duration of caspofungin combined with TMP/SMZ, days	-	-	13.9 ± 6.8	10.9 ± 5.3	0.052
Symptom onset to anti-PJ treatment, days	7.0[4.0, 9.5]	8.0[ 2.5, 10.5]	7.0[ 4.0, 10.0]	6.0[3.5, 9.0]	0.676
Symptom onset to ICU, days	8.0[5.0, 13.5]	8.0[4.0, 10.5]	9.0[6.0, 15]	7.0[ 4.0, 12.5]	0.058

SD, standard deviation; others*: aplastic anemia, poliomyelitis, thrombotic thrombocytopenic purpura, sequelae of cerebral hemorrhage, chronic cardiac insufficiency, cirrhosis

WBC, white blood cell; Neu, neutrophil; Lym, lymphocyte; PCT, procalcitonin; CRP, C-reaction protein; BDG, (1,3)-b-D-glucan; GM, galactomannan; LDH, lactate dehydrogenase; PJ, Pneumocystis jirovecii

### Outcome in all patients

In the study, the median (IQR) time of ICU stay was 16 days. A total of 43.01% of the patients received invasive mechanical ventilation due to respiratory failure. The overall positive response rate to PCP treatment was 59.13% (n = 55), and the overall 90-day mortality rate was 46.23%. The clinical outcomes of the cohort at the end of PCP treatment are shown in Table 2.

**Table 2:** Clinical outcomes of the cohort at the end of pneumocystis pneumonia (PCP) treatment

variables	All (n = 93)	Group1(n = 21)	Group2(n = 43)	Group3(n = 29)	*P* value
Invasive mechanical ventilation [n (%)]	40(43.0%)	11(52.4%)	14(32.6%)	15(51.7%)	0.168
shock	33(35.5%)	7(33.3%)	16(37.2%)	10(34.5%)	0.946
Length of ICU stay(days)	16.0[10.0, 28.0]	24.0[ 11.5, 35.0]	14.0[8.0, 28.0]	18.0[12.5, 25.5]	0.245
Positive response rate[n (%)]	54(58.1%)	11(61.9%)	33(76.7%)	10(34.5%)	0.001
90-day mortality [n (%)]	46(49.5%)	10(48.6%)	17(39.5%)	19(65.5%)	0.095

The survival rates of the patients in each group were 51.38%, 60.47% and 34.49%, respectively.

The patients who received initial combination therapy with caspofungin and TMP/SMZ (Group 2) had a better positive response rate than those in group 1 and group 3, and the difference was statistically significant (76.74% vs. 61.90%, 34.49%, *p* = 0.001). The patients who received initial combination therapy had a lower 90-day mortality rate than those in group 1 and group 3, but there was no significant difference (39.53% vs. 48.62%, 65.51%, *p* = 0.095).

In univariate analysis, factors associated with positive response rate: Aspergillus co-infection (OR 0.232, 0.07–0.81, *p* = 0.022), invasive mechanical ventilation (OR 0.32, 0.14–0.75, *p* = 0.009), and anti-PCP regimens (OR6.27,2.21–17.78, *p* = 0.001). Only anti-PCP at admission (OR 5.87, 1.87–18.38; *p* = 0.002) was independently associated with positive response rate (Table 3).

**Table 3:** Univariate and multivariate analyses of factors associated with positive responses to therapy

	Univariate analysis(n = 93)			Multivariate analysis(n = 93)		
	OR	95%CI	p-value	OR	95%CI	p-value
Therapeutic regimens						
Anti-PCP	6.27	2.21–17.78	0.001	5.87	1.87–18.38	0.002
Co-infections						
Pulmonary aspergillosis	0.23	0.07–0.81	0.022	0.33	0.08–1.42	0.136
Bacteremia	0.39	0.14–1.08	0.069	0.90	0.27–3.01	0.862
Invasive mechanical ventilation	0.32	0.14–0.75	0.009	0.40	0.14–1.11	0.077

According to different Anti-PCP treatments: SMZ/TMP as monotherapy; initial caspofungin combined with TMP/SMZ; caspofungin combined with TMP/SMZ as salvage therapy

**Fig. 1:**
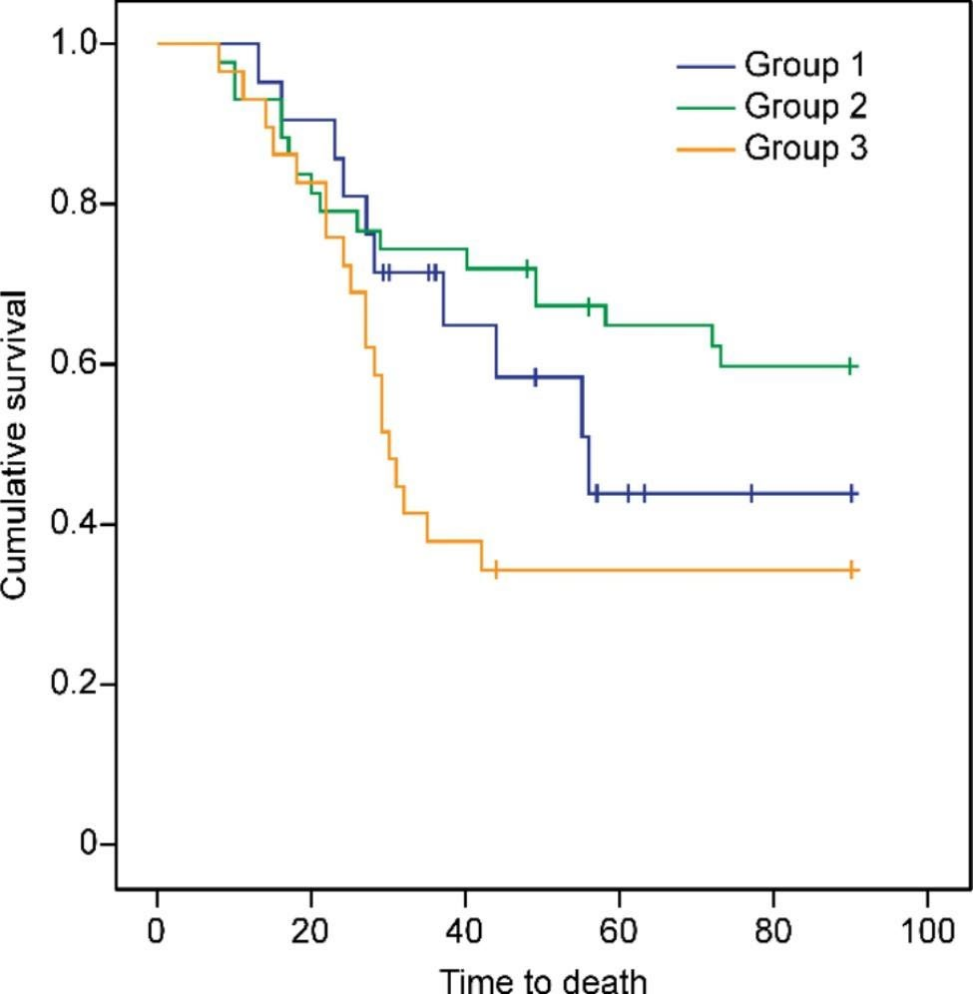
Kaplan‒Meier survival plot for 90 days of follow-up regarding PCP-related mortality

The Kaplan‒Meier curve indicated that there were no statistically significant differences in the survival rate among the three groups of patients, and caspofungin combined with TMP/SMZ initially had higher survival rates during the hospital stay (60.46% vs. 52.38%, 34.48%, *p* = 0.095) (Fig. 1).

### Incidence of adverse events

Adverse events occurred in 28 patients (30.11%) and included erythrocytopenia (8.6%), leukocytopenia (8.6%), thrombocytopenia (13.98%), elevated liver enzymes (10.5%), renal dysfunction (18.28%), nausea and vomiting (5.38%) and drug eruption (3.23%). There were no significant differences between the three groups. There were no serious events caused by caspofungin therapy.

## Discussion

Our results demonstrated that the positive response rate of patients who received initial caspofungin combined with TMP/SMZ was 72.09%, which was higher than those who received TMP/SMZ monotherapy and those who received combination therapy as salvage treatment.

Pneumocystis exists in three morphologically distinct forms: the trophic form, the predominate form and the cyst form [17]. Only the cyst form of pneumocystis species has beta-(1,3)-D-glucan. Caspofungin is theoretically feasible and competitively inhibits the formation of Pneumocystis through inhibition of fungal cell-wall synthesis but it does not appear to be effective enough to achieve a cure as a sole therapy alone [18]. Over the past decade, caspofungin has often been used as salvage therapy only after TMP-SMZ failure or intolerance [19]. However, it is difficult to draw conclusions on its efficacy due to the limited data [20]. There are only a few reports suggesting that SMZ plus caspofungin is effective [21, 22]. In our study, we compared the difference in positive response rate and 90-day all-cause mortality between the treatment regimen of combination therapy initially and the shift regimen as salvage therapy. We found that there was an advantage of caspofungin and TMP/SMZ as initial therapy compared to other treatment regimens. A possible explanation for this might be the ability of caspofungin to slow or even stop PCP growth via consistent depletion of the fungal burden [23]. In animal models, caspofungin was indicated to competitively inhibit the formation of Pneumocystis through inhibition of fungal cell-wall synthesis [8, 24, 25]. Another reason for this might be that non-HIV patients progress more rapidly and severely with higher inflammation in the lungs than HIV-infected patients [26–28]. Therefore, the timeliness of early treatment is particularly important. TMP/SMZ has a slow onset of action, and caspofungin acts faster [29], which could have a complementary anti-PCP effect in the early stage of the disease. In our study, compared with combination initially, the patients who had a combination as salvage therapy showed poorer response and survival rates.

Our findings showed a tendency of the initial combination therapy to improve the outcomes for immunocompromised patients with PCP. However, we did not find significant differences in the survival rate among the three groups. We speculate that this might be because the patients we enrolled presented a more severe clinical picture with coinfections and invasive mechanical ventilation. Inconsistent with previous studies, our study showed a higher coinfection rate in approximately 74.19% of the patients, among whom 15.05% (n = 14) had pulmonary aspergillosis and 23.65% (n = 22) had CMV infections. In the previous literature, coinfections are considered indicators of poor prognosis, particularly when CMV infection or pulmonary aspergillosis develops [30, 31]. Additionally, nearly half (43.01%) of the enrolled patients required invasive mechanical ventilation, with an average oxygenation index of 113.02 ± 53.09. Boonsarngsuk’s study [32] showed that the mortality rates do not differ widely, especially when the disease progresses to acute respiratory failure requiring mechanical ventilation. Finally, we found that the group that received monotherapy had lower serum BDG levels than the combination groups. Although previous studies have not found a correlation between serum BDG and disease severity, BDG levels can reflect the fungal load in the lungs [33, 34]. We speculate that this may also be related to the lack of a significant difference in mortality with early combination therapy compared with monotherapy.

There is still a need for more in-depth research, as an increasing number of individuals are being immunosuppressed for autoimmune diseases and organ transplantation.

Importantly, in terms of drug side effects, none of the patients had serious adverse events from caspofungin therapy, which is consistent with a previous study [35]. However, the combination therapy of caspofungin and TMP/SMZ does not increase the incidence,

There were several limitations to the present study. First, the clinical data from the patients in the study were retrospectively collected, and inherent biases were inevitable. Second, the sample size was relatively small, and only 41.9% of the patients had alveolar lavage fluid or blood PCR results. The fungal load was not quantified by PCR.

In conclusion, for non-HIV-infected patients with severe PCP, initial combination therapy with caspofungin and TMP/SMZ is a promising and relatively safe treatment option compared with TMP/SMZ monotherapy and combination therapy as salvage therapy. Our study findings offer guidance to clinicians in the early management of patients with severe PCP.

## Data Availability

The datasets generated and/or analyzed during the current study are not publicly available due individual privacy of patients could be compromised, but are available from the corresponding author on reasonable request.
